# Advancements of MOFs in the Field of Propane Oxidative Dehydrogenation for Propylene Production

**DOI:** 10.3390/molecules29061212

**Published:** 2024-03-08

**Authors:** Shu-Ting Li, Ming Ke, Jie Zhang, Yun-Lei Peng, Guangjin Chen

**Affiliations:** 1 Department of Applied Chemistry, College of Science, China University of Petroleum-Beijing, Beijing 102249, China; 2022211349@student.cup.edu.cn (S.-T.L.); keming_0824@163.com (M.K.); 2 State Key Laboratory of Heavy Oil Processing, China University of Petroleum-Beijing, Beijing 102249, China; 3 Basic Research Center for Energy Interdisciplinary, China University of Petroleum-Beijing, Beijing 102249, China; 4 Tianjin Institute of Industrial Biotechnology, Chinese Academy of Sciences, Tianjin 300308, China; 15242308736@163.com

**Keywords:** metal-organic frameworks, propane oxidative dehydrogenation, propylene, propane

## Abstract

Compared to the currently widely used propane dehydrogenation process for propylene production, propane oxidative dehydrogenation (ODHP) offers the advantage of no thermodynamic limitations and lower energy consumption. However, a major challenge in ODHP is the occurrence of undesired over-oxidation reactions of propylene, which reduce selectivity and hinder industrialization. MOFs possess a large number of metal sites that can serve as catalytic centers, which facilitates the easier access of reactants to the catalytic centers for reaction. Additionally, their flexible framework structure allows for easier adjustment of their pores compared to metal oxides and molecular sieves, which is advantageous for the diffusion of products within the framework. This property reduces the likelihood of prolonged contact between the generated propylene and the catalytic centers, thus minimizing the possibility of over-oxidation. The research on MOF catalyzed oxidative dehydrogenation of propane (ODHP) mainly focuses on the catalytic properties of MOFs with cobalt oxygen sites and boron oxygen sites. The advantages of cobalt oxygen site MOFs include significantly reduced energy consumption, enabling catalytic reactions at temperatures of 230 °C and below, while boron oxygen site MOFs exhibit high conversion rates and selectivity, albeit requiring higher temperatures. The explicit structure of MOFs facilitates the mechanistic study of these sites, enabling further optimization of catalysts. This paper provides an overview of the recent progress in utilizing MOFs as catalysts for ODHP and explores how they promote progress in ODHP catalysis. Finally, the challenges and future prospects of MOFs in the field of ODHP reactions are discussed.

## 1. Introduction

Propylene, as one of the most prominent industrial raw materials, holds significance in terms of demand, second only to ethylene. The global propylene market is estimated to reach 117.5 million metric tons in 2022 and is projected to reach a revised volume of 155.2 million metric tons by 2030, exhibiting a compound annual growth rate of 3.5% during the analysis period from 2022 to 2030 [1]. DHP is a significant method for the production of C_3_H_6_ in industry. However, due to the unfavorable thermodynamics of DHP, the reaction temperature generally exceeds 500 °C, leading to catalyst deactivation caused by carbon deposition. This difficulty hinders the development of the DHP process [2]. In comparison with DHP, ODHP is not limited by thermodynamics. It introduces oxidants such as O_2_ into the reaction system, enabling the reaction to occur at lower temperatures. Additionally, it allows for the rapid oxidation of carbon deposits on the catalyst’s surface during the reaction, thus avoiding catalyst deactivation and effectively prolonging the catalyst’s lifespan [3]. However, due to the lower average C-H bond energy of propylene compared to propane, propylene is prone to over-oxidation, resulting in the generation of CO_x_ during ODHP. Therefore, an ideal catalyst for ODHP needs to effectively suppress the over-oxidation of propylene while activating propane.

Typical catalysts for ODHP are supported by vanadium, molybdenum, and chromium oxides. These catalysts often exhibit good catalytic activity but poor selectivity [3]. The catalytic activity is influenced by the density of active sites on the catalyst surface, with a low density of active sites leading to propylene coming into contact with the support and undergoing over-oxidation. Conversely, complete coverage of the substrate with well-dispersed active species enables high selectivity towards propylene. However, an excessively high density of active species, such as vanadium, exhibits a propensity for polymerization, which favors continuous propylene combustion reactions and leads to decreased selectivity for propylene [4]. Furthermore, as the identity and morphology of the support and catalyst species play a crucial role in catalyst improvement, the enhancement of ODHP catalysts is largely empirical [5]. This highlights the urgent need to address how to maintain high catalytic rates while ensuring high selectivity towards olefins, how to achieve controllable modulation of catalyst structure, and how to elucidate the catalytic mechanism of active species.

MOFs are a new class of porous crystalline materials composed of metal ions coordinated with organic ligands that exhibit periodic, multidimensional network structures. They possess features such as large specific surface areas, flexible and adjustable structure-functionality, etc. [6,7,8]. Due to the tunability of organic ligands in MOFs, the variable coordination number of central metal atoms, and the diversity of connectivity with ligands, the pore size and shape of MOFs can be conveniently adjusted. These distinct pores can selectively accommodate reactants and products of catalytic reactions based on their size and even shape. Furthermore, the tunability of MOFs through the selection of organic ligands containing specific functional groups allows for the preparation of functionalized MOFs with these groups, the presence of which may influence the yield or selectivity of catalytic reactions [9]. At the same time, the high dispersion of active sites resulting from the ordered structure of MOFs can prevent performance decay caused by agglomeration when loading a large amount of active species [10]. Moreover, the periodic order of MOFs provides an excellent platform for studying the catalytic mechanism of active species, allowing for the construction of a series of MOFs to investigate their structure–activity relationship, clarify the operating mechanism, and further regulating the catalytic activity of catalysts [11]. These unique advantages make MOFs a hot research topic in the field of catalysis. However, current research on MOF materials for catalyzing ODHP has not received sufficient attention. Therefore, summarizing the current research progress and proposing improvements in the catalytic performance of MOF materials for ODHP methods are highly important and urgent (Table 1).

This paper presents the first comprehensive review of the development of MOF-based catalysts for ODHP and is especially concerned with the mechanism of reaction. In addition, we further reviewed the synthesis, characterization, and performance evaluation methods of MOF-based catalysts. Finally, we state the urgent challenges that need to be overcome for their industrial application. As this first review specifically summarizes ODHP with MOF-based catalysts, it is destined to play an important enlightening role for future research.

## 2. The Research Status of MOF-Based Catalysts in Catalyzing ODHP

**Figure 1:**
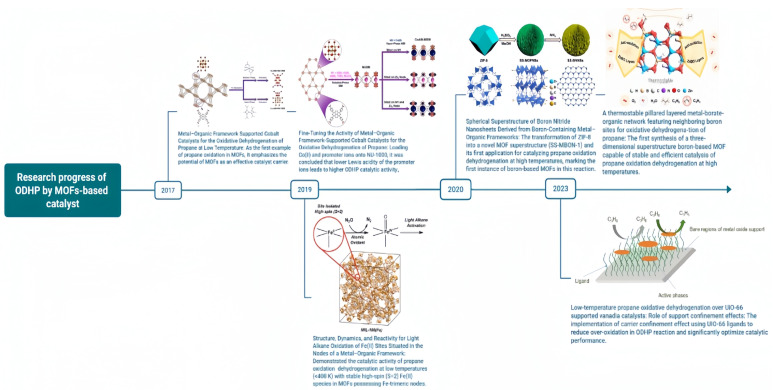
Research progress of ODHP by MOF-based catalysts.

Compared with commonly used and supported metal oxide catalysts in ODHP, MOF-based catalysts are a novel class of catalysts that have only been introduced into the field of ODHP research in 2017 (Figure 1). In contrast to mono-active site metal oxide catalysts, MOF-based catalysts exhibit diverse catalytic active species and demonstrate superior catalytic performance in ODHP (Table 2). The in situ-generated oxidized active species play a pivotal role in achieving high propane conversion rates.

Furthermore, an important issue to be addressed in ODHP is the over-oxidation of propylene [3]. Effective methods to enhance propylene selectivity include regulating the redox potential of the metal center serving as the active site [12] and controlling the contact between propylene and the metal center through the formation of organic ligand barriers [3] or the modulation of the pore structure. These strategies can be easily achieved by the targeted modification of MOF-based materials.

### 2.1. Catalytic ODHP by MOF-Supported VO_x_

**Figure 2:**
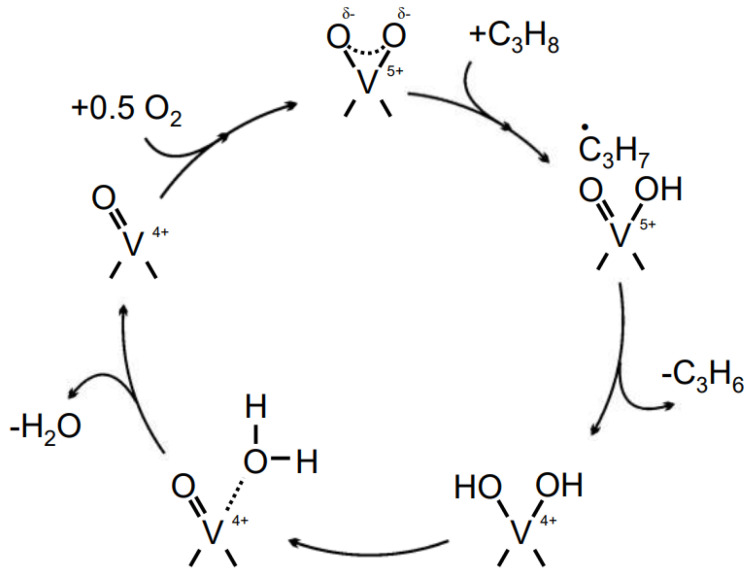
Catalytic cycle for propane ODH over V^5+^ − O^−^ radical site [3]. Copyright 2015, Berlin, Germany, Springer Nature.

Vanadium-based catalysts that are supported on carriers have been extensively researched for their ability to facilitate propane oxidative dehydrogenation (ODH) and are considered to be among the most promising materials with ODHP catalytic activity [25,26]. These catalysts operate according to the Mars–van Krevelen mechanism, which involves lattice oxygen. This catalytic process takes place on the well-recognized radical active sites on the catalyst surface during propane ODH (refer to Figure 2). Initially, the surface oxygen radical takes a hydrogen atom from propane, creating a C_3_ radical and saturated V^5+^. Afterwards, V^5+^=O reacts with the C_3_ radical to produce the released propylene and the hydroxylated reduced vanadium species V^4+^. Finally, the hydrogen atom on the reduced hydroxyl moves to another hydroxyl, forming adsorbed water, which is then hydrolyzed [3]. Once desorbed, V^4+^ is re-oxidized by oxygen atoms, resulting in the regeneration of active sites.

**Figure 3:**
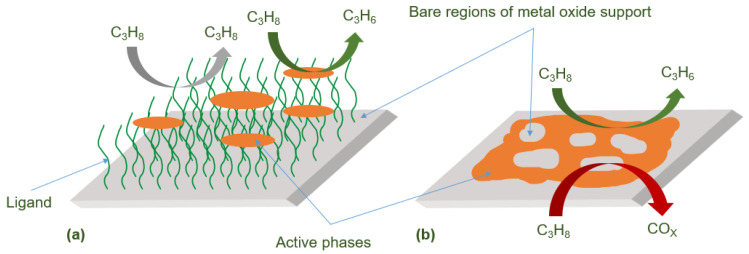
Conceptual representation of (**a**) support-confined catalyst system and (**b**) traditional supported catalyst system [15]. Copyright 2020, New York, NY, USA, American Chemical Society.

Numerous studies on supported vanadium-based catalysts have indicated that the catalyst activity depends on the properties of the carrier and the structure of the surface VO_x_ material [27,28,29]. It is the isolated tetrahedral VO_x_ species that play a role in selectively producing propylene. This indicates that one of the important factors influencing the performance of vanadium-based catalysts is the loading quantity of active VO_x_ sites. When the loading quantity is low, the active species are insufficient to cover the surface, leading to the over-oxidation of propylene on the carrier surface (Figure 3b) resulting in reduced propylene selectivity [4]. On the other hand, when the loading quantity is high, VO_x_ tends to aggregate and form V_2_O_5_ particles, which possess strong oxidative properties, leading to decreased propylene selectivity. This implies that achieving high dispersion while reducing the over-oxidation of propylene is a key challenge in improving supported vanadium-based catalysts.

Currently, the main carriers used for vanadium-based catalysts are SiO_2_, Al_2_O_3_, and other oxide carriers. However, traditional oxide carriers have low surface areas, making it difficult to achieve high loading and dispersion of active species [30]. The high specific surface area of the catalyst will enable active species to achieve high loading while minimizing the formation of V_2_O_5_ particles due to aggregation, thereby ensuring catalytic activity, a feat that is difficult to achieve with low specific surface area catalysts; therefore, researching carriers with high specific surface areas is an inevitable direction for improving the performance of vanadium-based catalysts. Researchers have conducted extensive studies on zeolites as carriers for ODHP active phases. For example, Solsona et al. [31] prepared high specific surface area layered material ITQ-6 as a carrier, while Karakoulia et al. [32] used MCM-41, HMS, and SBA-15 as carriers. These studies have demonstrated that, under the same vanadium loading quantity, zeolites exhibit superior dispersion of vanadium species and catalytic performance compared to the traditional oxide carrier SiO_2_. Compared to zeolites, MOFs not only possess ultra-high specific surface areas, but also offer the possibility of adjusting catalytic performance by changing the types of metal ions and the properties of coordinating groups [6,7,8], making them potential carriers for ODHP active phases.

Among the most commonly used MOFs, UiO-66 is constructed from zirconium oxide nodes connected by 12 1,4-benzenedicarboxylate ligands [15]. The abundant ligands act as barriers to prevent propylene from accessing the zirconium oxide nodes (Figure 3a). Farzaneh et al. [15] selected UiO-66 as a carrier for vanadium salts and obtained a vanadium loading of 5.97% in 6V/UiO-66. Compared to previously reported supported vanadium catalysts [17,18,19,20,21,33,34], this composite material exhibits a lower operating temperature and vanadium species density, but higher propylene productivity. These results indicate that using UiO-66 as the carrier not only provides a high specific surface area platform for the dispersion of VO_x_ species but also that the abundant organic ligands act as barriers, reducing the necessary amount of vanadium salts for covering the carrier surface and achieving a higher density of selectively isolated vanadium species, which has a favorable impact on propylene selectivity.

Although 6V/UiO-66 exhibits excellent catalytic performance, there is still a risk of vanadium species detachment since the vanadium species are only dispersed on the surface of UiO-66. Vanadium-based MOFs possess the unique ability to simultaneously combine the excellent redox properties of vanadium with the strong tunability of MOFs’ crystalline structures. By introducing auxiliary ligands or additional metals, the spatial and electronic properties of vanadium metal sites can be adjusted, thereby changing their catalytic performance. Exploring the possibility of using vanadium-based MOFs as ODHP catalysts can be considered as the next step in improving MOF-based vanadium catalysts.

### 2.2. Catalytic Low-Temperature ODHP by Co(II/III) Species

Although ODHP offers a lower reaction temperature compared to direct dehydrogenation, conventional vanadium and molybdenum-based catalysts still require relatively high operating temperatures (>500 °C) [22]. This often leads to extensive conversion of the desired product, such as C-C bond cleavage. In contrast, cobalt oxide has been used due to its ability to catalyze reactions under mild conditions. Previous studies have shown that nanostructured cobalt oxide can activate propane to produce propylene at room temperature [35], but with rapid deactivation, low conversion rates, and an unclear catalytic mechanism.

Huang et al. [22] synthesized layered double oxides (LDOs) of Co-Al and used them as catalysts for ODHP, demonstrating that the low-temperature catalytic behavior in ODHP is actually due to the reduction of Co(III)-related species. Cai et al. [36] prepared NiO-Co(x) composite oxides and used them for low-temperature propane oxidation, further indicating that the activity of supported cobalt catalysts is dependent on the cobalt species content and that the main catalytic sites are located around or within the active oxygen of nanostructured Co_3_O_4_. These studies suggest that controlling the quantity of cobalt oxide species and elucidating the catalytic mechanisms of cobalt oxide species are crucial for improving cobalt-based catalysts.

**Figure 4:**
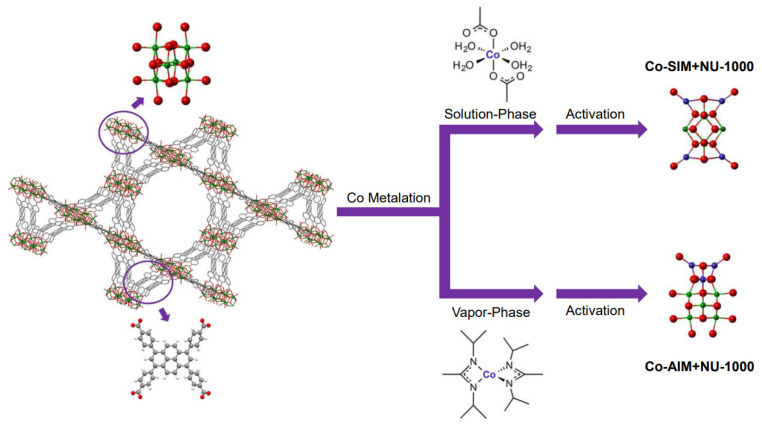
Structural representation of the MOF support, NU-1000, as well as the two preparation methods of Co-based catalysts for propane ODH catalysis [11]. Copyright 2016, New York, NY, USA, American Chemical Society.

**Figure 5:**
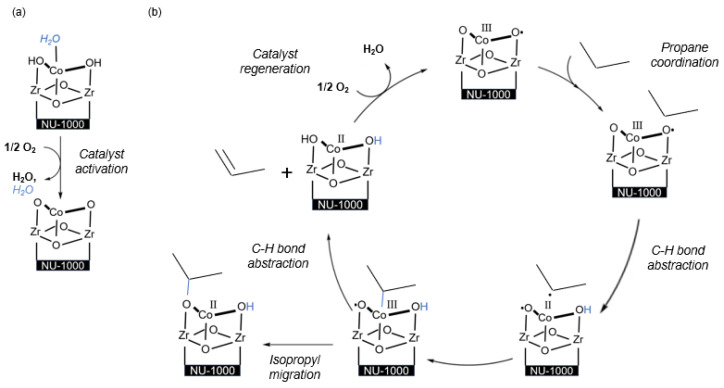
(**a**) Catalyst activation to form Co(III)-O· and (**b**) computed ODH mechanism using the activated species Co(III)-O· [11]. Copyright 2016, New York, NY, USA, American Chemical Society.

MOFs have strong structural modifiability and well-defined crystal structures, making them ideal for studying the mechanism of catalysis when used as supported catalysts. At the same time, NU-1000, a Zr-based MOF with good thermal stability, has structurally defined −OH/−OH_2_ groups on its Zr6 nodes, which can act as site-isolating grafting sites to control the deposition of metal ions. It has been reported as an almost Ideal carrier for Ir-based and Ni-based gas-phase catalysts [37,38]. Li et al. [11] first applied MOFs to the catalytic ODHP field, using two deposition techniques to anchor Co (II) ions to NU-1000, namely solvent thermal deposition (SIM) and atomic layer deposition (AIM) in the MOF (Figure 4). They demonstrated that the prepared Co-AIM+NU-1000 and Co-SIM+NU-1000 both exhibited catalytic activity for propane ODH at temperatures as low as 200 °C, whereas commonly used ODHP catalysts with V-oxo sites require temperatures of 300–500 °C. Additionally, both achieved maximum conversion rates at 230 °C and maintained catalytic activity for 24 h, with their turnover frequency (TOF) surpassing that of highly dispersed cobalt catalysts on zirconium oxide. Co-AIM+NU-1000 exhibited better catalytic activity than Co-SIM+NU-1000, with the spectroscopic characteristics of cobalt oxygen clusters and cobalt mixed valence similar to the spinel Co_3_O_4_ structure. Density functional theory (DFT) calculations using a single cobalt species model showed that the catalytic activity of Co-SIM+NU-1000 is derived from the conversion between Co(II)-OH and Co(III)-O· (Figure 5). Co(II)-OH reacts with oxygen to generate the active species Co(III)-O· and forms water, and then, Co(III)-O· extracts hydrogen atoms from propane to generate C_3_ radicals. These C_3_ radicals can combine with the cobalt center to form more stable intermediates, which can either extract hydrogen atoms from C-H bonds to generate propylene and the reduced form of Co(II)-OH or undergo isopropyl migration to form rebound intermediates. Calculations of the reaction enthalpy for Co(III)-O·-catalyzed ODHP showed that at higher temperatures, isopropyl migration and the formation of rebound intermediates are more likely to occur, resulting in lower selectivity for propylene.

The active species of Co-SIM+NU-1000 is a cobalt(III)-oxygen species derived from cobalt(II) with coordination deficiencies [11]. This means that the catalytic activity of the oxygen–cobalt clusters loaded on MOFs as catalytic sites can be changed by controlling the redox potential of Co(II/III), and suitable heterometallic coordination is advantageous for changing the redox potential [39]. Li et al. [12] used Lewis acidic metal ions Ni(II), Zn(II), Al(III), Ti(IV), and Mo(VI) as additive agents to Co-AIM+NU-1000, and the activity of the resulting bimetallic oxide catalysts loaded on NU-1000 changed oppositely to the Lewis acidity of the additive ions. Differential envelope density (DED) analysis of synchrotron X-ray powder diffraction data and known single crystal X-ray structures of unmodified MOFs revealed that Mo(VI) did not interact with cobalt-oxygen clusters, and by simulating the relationship between the Lewis acidity of the additive ions and the catalytic activity (expressed as TOF) of the corresponding NU-1000 supported bimetallic oxides, it was found that only when the Lewis acidity of Co-Mo related to the oxygen-Zr(IV) node was assumed, the Lewis acidity and TOF were consistent with the presumed relationship, indicating that the Lewis acidic metal ions that actually play a role are Zr^4+^ with the maximum interaction with the active site oxygen–cobalt clusters, rather than Mo(VI). This suggests that the key factor in changing the catalytic performance of the additive ions is their Lewis acidity relative to the oxygen–cobalt active site.

Based on the understanding that the catalytic activity of the cobalt species loaded on MOFs originates from the conversion between Co(II)-OH and Co(III)-O· [11], it becomes possible to directly use cobalt-based MOFs capable of forming coordination unsaturated oxygen–cobalt species as ODHP catalysts. This is easier to achieve than anchoring cobalt ions to the metal nodes of MOFs, and the regulation of the catalytic activity of the oxygen–cobalt species in cobalt-based MOFs can be achieved by introducing additive ions with weaker Lewis acidity. This indicates that further research on cobalt-based MOFs in the catalytic performance of ODHP reactions can further advance the development of low-temperature ODHP catalysts.

### 2.3. Catalytic Low-Temperature ODHP by Fe(IV)-Oxo Species

The key ability of ODHP catalysts is to both break the non-polar C-H bonds in propane and effectively limit the overoxidation of propylene, which contains weaker C-H bonds [40]. In nature, iron metalloenzymes can activate dioxygen and easily activate strong C-H bonds through high-spin non-heme iron–carbonyl species, while controlling the access of substrates to the active site through complex channel environments to prevent overoxidation [41,42]. Scientists believe that simulating the function and properties of enzymes in solid supports is an effective method for designing lightweight alkane oxidation catalysts, and MOFs, with their porous structures and various components and topologies, are ideal carriers for iron sites for alkane activation. Xiao et al. [43] oxidized Fe(II)-containing Fe-MOF-74, which has unsaturated coordination, with the commonly used two-electron oxidant N_2_O, and found the formation of Fe(III)-OH species, indicating the generation of Fe(IV)=O intermediates. Subsequently, Fe-MOF-74 was reacted in a mixture of N_2_O, ethane, and Ar, and it was found to have the ability to oxidatively dehydrogenate ethane to ethanol, demonstrating the potential production of high-spin non-heme iron(IV)–oxygen species capable of activating the strong C-H bonds of ethane under N_2_O activation. Verma et al. [44] further explored the mechanism of ethane oxidation to ethanol catalyzed by Fe-MOF-74 and proposed ethyl radical rebound as a pathway for ethanol formation. After exploring competing pathways, it was concluded that hydroxylation is more favorable compared to desaturation and dissociation in the catalytic oxidation of ethane by Fe-MOF-74. The elucidation of the mechanism of ethane oxidation and dehydrogenation catalyzed by Fe-MOF-74, which has unsaturated Fe(II) species oxidized by N_2_O to form Fe(IV)=O intermediates, makes it possible for more MOFs with unsaturated Fe(II) species to exhibit the ability to activate strong C-H bonds upon N_2_O oxidation. Osadchii et al. [45] incorporated Fe into MIL-53(Al) to prepare MIL-53(Al, Fe) and found that it can catalyze the oxidative dehydrogenation of methane to methanol using H_2_O_2_ as the oxidant. They proposed that the activity of MOFs in the partial oxidation of light alkanes requires isolated, high-spin (S = 2), antiferromagnetically coupled iron sites.

Although many studies have investigated the reactivity and mechanism of methane and ethane oxidation and dehydrogenation catalyzed by MOFs with coordinatively unsaturated iron(II) species, there has been little research on the reactivity of related MOFs in propane oxidation and dehydrogenation. It has been confirmed that high-spin Fe(IV)=O species can activate strong C-H bonds, which makes it possible for them to catalyze the oxidation and dehydrogenation of propane.

**Figure 6:**
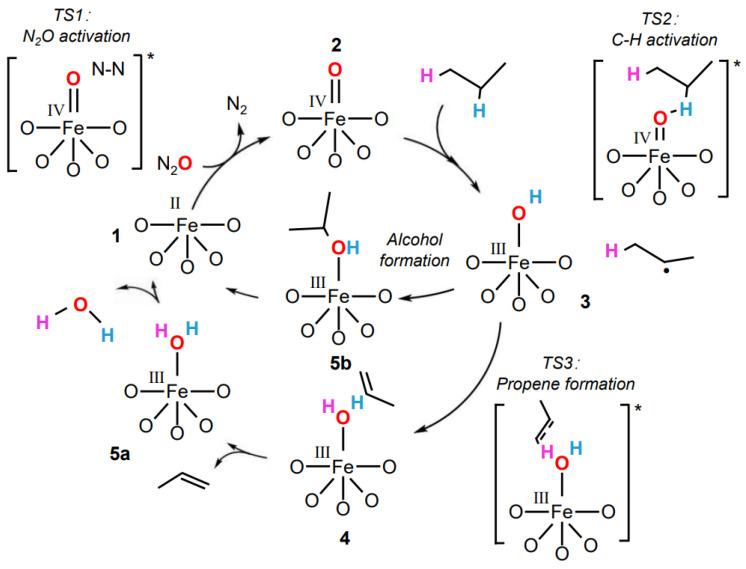
Reaction scheme for the oxidative dehydrogenation of propane to propylene and the selective oxidation of propane to 2-propanol using N_2_O as the oxidant [49]. The numerals indicate the sequence of reactions, while arrows represent the direction of activation and regeneration of active sites. Asterisks (*) intermediates that are unstable or undergo rapid transformation during the reaction process, and dots imply the presence of free radicals. Copyright 2019, New York, NY, USA, American Chemical Society.

MIL-100(Fe) is a MOF composed of trimeric Fe(III)-oxo nodes and a benzene-1,3,5-tricarboxylic acid-derived linker. It can form coordinatively unsaturated and high-spin (S = 2) Fe(II) species after thermal treatment [46,47,48]. This meets the requirements for catalytically active sites as proposed by Osadchii et al. [45], and it is speculated by Simon et al. [13] that it may also have the ability to activate light alkanes after N_2_O oxidation. Therefore, Matthew et al. first applied MIL-100(Fe) to the propane oxidation dehydrogenation reaction, and it was found that MIL-100(Fe), after vacuum pre-treatment at 523 K, can catalyze the oxidation and dehydrogenation of propane to propylene using N_2_O as the oxidant at 378 K. The common mechanism for the activation of alkanes by Fe-oxo species is radical rebound to form hydroxylated products [43,44]. However, in the products of propane oxidation and dehydrogenation catalyzed by MIL-100(Fe), the yield of propylene is higher than that of propanol. Therefore, a new secondary C-H abstraction mechanism, confirmed by DFT calculations, has been proposed (Figure 6). In this mechanism, the unsaturated Fe(II) species is oxidized by N_2_O to form Fe(IV)=O intermediates, which then extract hydrogen atoms from propane and radicals, respectively, and eliminate water to generate propylene. The Fe(II) is then regenerated and continues the catalytic cycle. In addition, DFT calculations show that the antiferromagnetic coupled clusters in the trimeric nodes are the reason for the activity of MIL-100(Fe). It emphasizes that the stability of the high-spin (S = 2) Fe(II) species in MIL-100(Fe) is due to the weak ligand field associated with the surrounding O atoms, which allows it to react with N_2_O at low temperatures and activate light alkanes. Therefore, the stability of the high-spin (S = 2) Fe(II) species is the key to activating light alkanes at low temperatures.

The studies on the active sites and reactivity of MIL-100(Fe), as well as the proposal of the Fe-oxo secondary C-H abstraction mechanism [13], have demonstrated that MOFs with trimeric Fe-oxo nodes, including MIL-100(Fe), have sites capable of activating light alkanes. This provides a new direction for designing ODHP catalysts.

Barona et al. [49] showed that the binding energy of oxygen to metal sites is inversely proportional to the ability of MOFs to activate strong C-H bonds [50]. Based on the fact that N_2_O decomposition and C-H bond cleavage are the two energetically demanding steps on the trimeric Fe-oxo nodes for Fe(II)-catalyzed light alkane oxidation and dehydrogenation, they systematically modified the composition of the metal nodes (Fe_2_M) in PCN-250 and used DFT calculations to evaluate their catalytic performance for propane ODH with N_2_O as the oxidant. The results showed that weaker oxygen binding energy to the metal center leads to lower N_2_O activation barriers but higher C-H activation barriers. Specifically, when M is an early transition metal like V or Ti, the metal center has strong oxygen binding, which favors N_2_O activation but hinders C-H activation. Experimental validation confirmed that PCN-250(Fe_2_Mn) and PCN-250(Fe_3_) exhibit better catalytic activity than PCN-250(Fe_2_Co) and PCN-250(Fe_2_Ni), consistent with the DFT calculations.

Based on the understanding of the correlation between oxygen binding energy at the metal center and N_2_O decomposition and C-H bond cleavage, further exploration of the activity of MOFs containing trimeric Fe-oxo nodes for propane oxidation and dehydrogenation is made possible through DFT calculations and experimental validation. This provides a convenient approach for improving MOF-based ODHP catalysts.

### 2.4. Improvement of ODHP Boron-Based Catalysts with Three-Dimensional Spherical Superstructure MOFs

**Figure 7:**
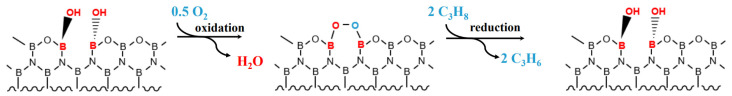
Proposed redox reaction cycle in the oxidative dehydrogenation of propane over boron nitride [55]. Copyright 2018, Dalian Institute of Chemical Physics, Beijing, China, Chinese Academy of Sciences.

Following the serendipitous discovery of hexagonal boron nitride (h-BN) as a breakthrough catalyst for ODHP [51], Shi et al. [24] conducted edge hydroxylation of boron nitride and observed that the hydroxylated form exhibited higher alkene yields compared to h-BN. Based on infrared spectroscopy and ^18^O isotope tracing, they proposed that the B-OH groups on the edges of boron nitride were initially oxidized by molecular oxygen, followed by the promotion of propane dehydrogenation (Figure 7). In addition, Grant et al. [52] discovered that boron-containing materials such as elemental boron and boron carbide exhibited similar activity to BN in propane ODHP, while a nitrogen-containing catalyst, titanium nitride (TiN), used as a control, did not show comparable selectivity. This indicates that boron is the key element responsible for the ODHP activity exhibited by the catalyst. Based on X-ray photoelectron spectroscopy and infrared spectroscopy results, it was found that all boron-based materials displaying activity had similar stable BO_x_ active sites on their surfaces, suggesting that oxygen-containing boron sites play a crucial role in the unique catalytic functionality of boron-based materials in ODHP reactions [52]. This suggests that maximizing the number of oxygen-containing boron sites may be the most effective approach to enhancing the catalytic performance of h-BN. Furthermore, controlling the assembly of 2D h-BN nanosheets into 3D spherical superstructures proved to be an effective method for increasing the number of oxygen-containing boron sites at the edges of h-BN [53,54].

**Figure 8:**
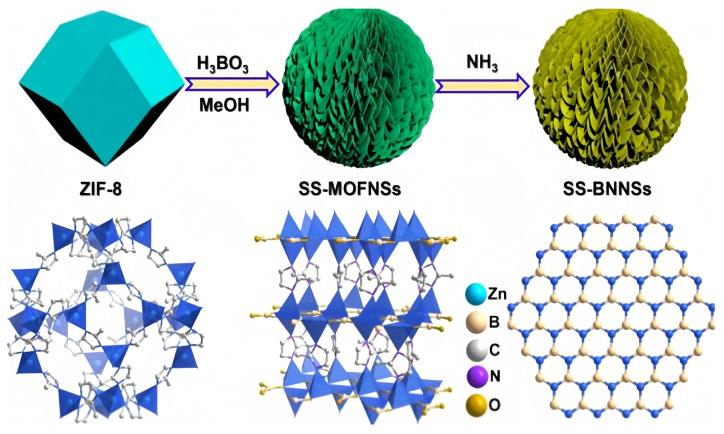
Schematic illustration of the creation of SS-MOFNSs and SS-BNNSs [14]. Copyright 2020, New York, NY, USA, American Chemical Society.

Using MOFs as sacrificial templates to prepare spherical superstructures is an effective strategy [56]. Cao et al. [14] synthesized a novel boron-containing MOF consisting of nanosheets (SSMOFNS) that could be converted into a three-dimensional spherical superstructure (SS-BNNS) composed of oxygen-rich boron nitride nanosheets with excellent catalytic performance under an ammonia atmosphere (Figure 8). The propylene yield obtained with this catalyst in the ODHP process was the highest among all h-BN-based catalysts. Both the pre- and post-reaction catalysts contained oxygen-functional groups, B-O and B-OH, indicating that SS-BNNS possessed active sites for ODHP catalysis from the beginning of its synthesis. Unlike h-BN, SS-BNNS could be used as a catalyst directly without the need for activation. Furthermore, the pore size distribution of SS-BNNS remained essentially unchanged before and after testing, demonstrating its structural stability and advantages over h-BN.

**Figure 9:**
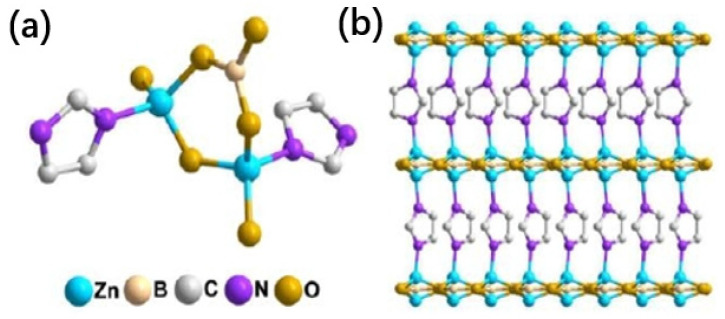
(**a**) Coordination environment of Zn atoms in MBON-2, (**b**) the whole structure of MBON-2 [2].

The preparation of SS-BNNS with improved ODHP catalytic performance provides a new approach for enhancing ODHP performance through structural engineering. The advantages of MOFs in preparing three-dimensional spherical superstructures are also demonstrated. However, the preparation conditions for SS-BNNS are quite stringent, requiring the nitridation of SSMOFNS in an ammonia atmosphere at 1000 °C. Directly preparing three-dimensional spherical superstructures with boron-rich oxygen sites in MOFs would theoretically be a simpler method. However, boron-based catalysts exhibit optimal catalytic performance for ODHP at temperatures above 490 °C, while MOFs often have poor high-temperature oxidation resistance and struggle to maintain their structure for stable catalysis under prolonged high-temperature conditions. Cao et al. [2] designed a novel boron-rich MOF called Metal Borate-Organic Framework-2 (MBON-2), in which B-OH functional groups were introduced into the MOF material (Figure 9). It was found that the pore size distribution and microstructure of MBON-2 remained essentially unchanged before and after the reaction, and it exhibited good high-temperature oxidation resistance, remaining stable up to 560 °C in an air atmosphere. This overcame the difficulties faced by traditional MOFs in catalyzing ODHP due to their poor high-temperature oxidation resistance.

**Figure 10:**
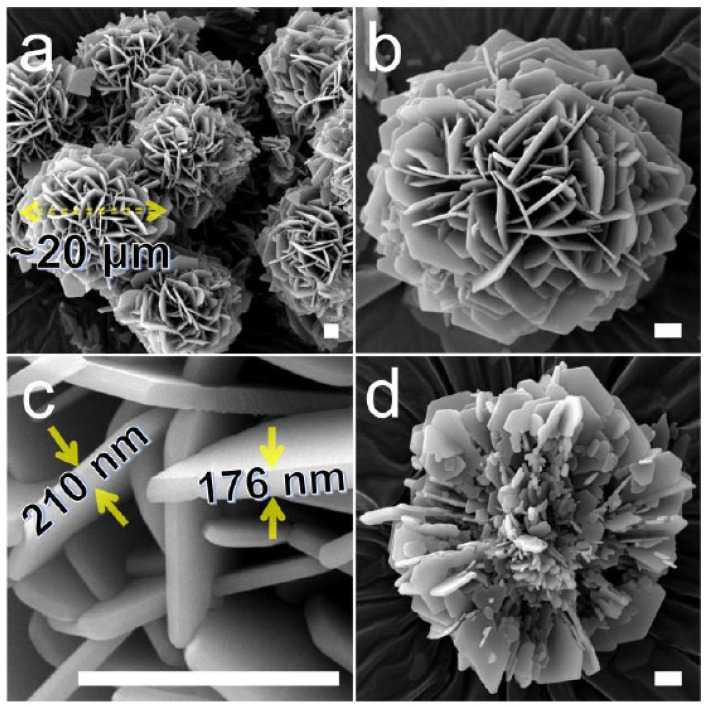
(**a**–**c**) SEM images of MBON-2. (**d**) SEM image of the internal structure of MBON-2. All the scales are 2 μm [2].

**Figure 11:**
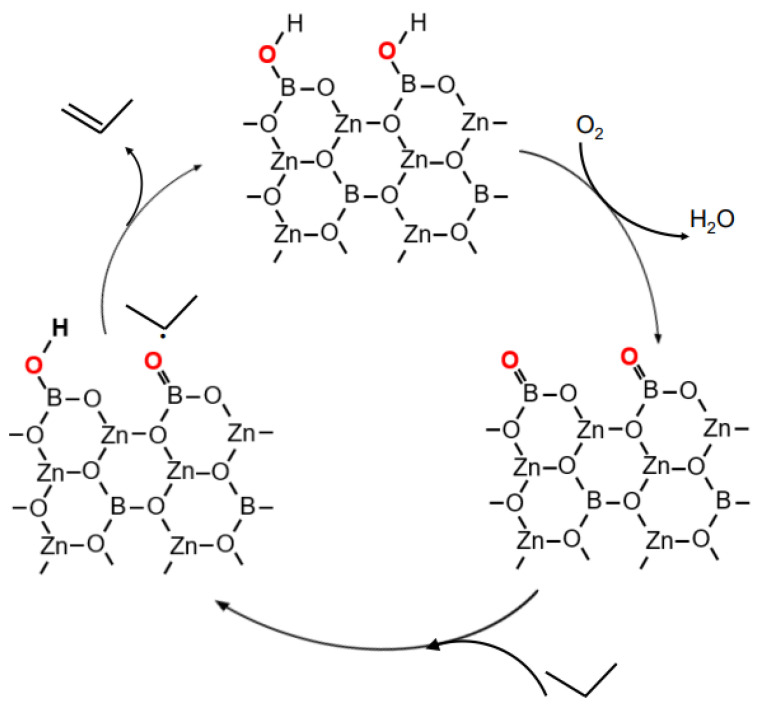
Proposed redox reaction cycle in the oxidative dehydrogenation of propane over MBON-2 [2]. Copyright 2023, Amsterdam, The Netherlands, Elsevier Inc.

MBON-2 is a three-dimensional floral superstructure composed of numerous MBON-2 lamellar materials assembled together (Figure 10). Compared to h-BN, it exhibits higher conversion rates. However, infrared in situ characterization and DFT calculations have shown that the catalytic activity of MBON-2 stems from the mutual conversion between B-OH and B=O on its edges (Figure 11). B=O possesses strong oxidizing properties, making it prone to causing over-oxidation of propylene and resulting in slightly lower selectivity compared to h-BN. Nonetheless, the advantage of MBON-2 lies in its easier synthesis, as it can be obtained through a low-temperature aqueous phase, highlighting its superiority over h-BN and SS-BNNS.

Currently, h-BN is one of the most efficient catalysts for ODHP, and it has been established that the oxygenated boron sites on its edges serve as the catalytic active sites. Developing catalysts with three-dimensional spherical superstructures to maximize the number of oxygenated boron sites is an effective strategy for improving the performance of boron-based ODHP catalysts. The successful synthesis of MBON-2, which exhibits comparable catalytic performance to h-BN, provides a direction for enhancing the performance of boron-based catalysts. However, despite the excellent catalytic performance of MBON-2, it does not exploit the catalytic activity of the abundant metal sites that are typically present as active centers in MOFs. Designing a new MOF that facilitates synergistic catalytic effects between the metal sites and boron oxygen sites would be an effective approach to improving the performance of boron-based catalysts.

## 3. Synthesis, Characterization, and Performance Evaluation of MOF-Based Catalysts

The development of MOFs in catalytic applications has been rapid, primarily due to the presence of high-density and uniformly dispersed catalytic active sites within the MOF structure. Additionally, the highly porous structure of MOFs ensures the accessibility of each catalytic active center. Moreover, the precise structure of MOFs allows for the elucidation of catalytic mechanisms at the active sites, leading to the derivation of a series of structure-mechanism MOF-based catalysts. Therefore, it is essential to thoroughly characterize the performance of MOF catalysts. This section will focus on the necessary steps involved in the study of MOF-based catalysts.

### 3.1. Synthesis

Various methods are commonly used for the synthesis of MOFs, including solvothermal synthesis, the diffusion method, the microwave method, etc.

(1) Solvothermal method [57]: This method is widely employed for MOF synthesis. In this method, polar solvents such as ethanol and water, ligands, and metal salts are mixed in certain proportions in a reaction vessel lined with polytetrafluoroethylene. The vessel is then sealed and transferred to an oven, where it is heated to create a high-temperature and high-pressure closed environment. This facilitates the dissolution of insoluble substances and promotes their reactions, leading to shorter reaction times. However, this method often employs organic solvents, which are not environmentally friendly or economically viable.
(2) Diffusion method [58]: This method is commonly used for the preparation of single crystals. In this method, organic ligands and metal salts are separately dissolved in two solvents of different densities. The solution containing the metal salt is placed above the solution containing the organic ligand, allowing the metal salt to diffuse into the lower solution under the force of gravity, leading to the formation of coordination compound crystals. This method enables the production of higher-quality crystals but requires the high solubility of reactants and is time-consuming.
(3) Microwave method [59,60]: This method is a novel approach for synthesizing MOF materials, utilizing microwave technology to induce high-frequency reciprocating motion of molecules within the heated substance. Unlike conventional heating methods, this approach does not require thermal conduction, thus enabling simultaneous heating of the interior and surface of the substance, ensuring uniform and efficient heating. Widely applied in organic synthesis, microwave-assisted synthesis has been found to be particularly advantageous in promoting nucleation rates and facilitating MOF growth, offering time- and energy-saving benefits compared to other synthesis methods. However, it presents challenges in separating large crystals and is not suitable for industrial-scale production of MOFs.
(4) Mechanochemistry synthesis [61,62]: Mechanochemical synthesis refers to a methodology where mechanical forces, such as compression, grinding, and shear, are employed to induce physical property changes and chemical reactions in reactants with minimal or no use of organic solvents. This approach does not require specific pressure or temperature conditions, but it presents challenges in isolating crystals suitable for X-ray single crystal diffraction.
(5) Electrochemical synthesis method [63,64]: This method utilizes electric energy to control and promote chemical reactions. It essentially involves electrolysis, where the metal ions generated at the anode during MOF synthesis react with organic ligands in the solvent to form coordination bonds, resulting in the formation of coordination compound crystals.

In addition, ionothermal synthesis [65], the solvent evaporation method [66], and the post-synthetic modification method [67] are also commonly employed in the synthesis, structural modulation, and performance improvement of MOFs.

### 3.2. Structure Characterization

**Figure 12:**
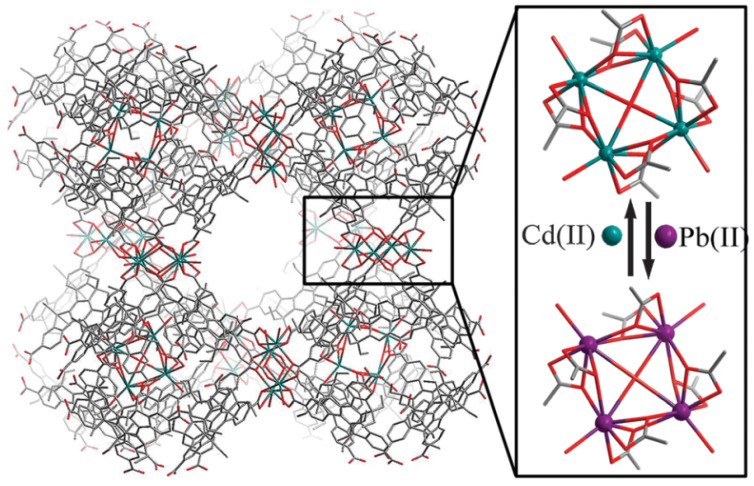
Post-synthetic metal ion exchange between isostructural frameworks [(Cd_4_O)_3_(hett)_8_] and [(Pb_4_O)_3_(hett)_8_]. Hydrogen atoms are omitted for clarity [69]. Copyright 2014, The Royal Society of Chemistry.

Characterization methods can be used to obtain specific information about MOF materials, such as structure and thermal stability. For example, the combination of high-temperature oxidation and XRD can be used to evaluate the high-temperature oxidative stability of MOF catalysts in high-purity air [2]. Nitrogen adsorption–desorption tests (BET) can provide information about the catalyst’s specific surface area, pore structure, and pore size distribution [68]. X-ray photoelectron spectroscopy (XPS) can be used to analyze the elemental distribution on the catalyst’s surface and changes in functional groups before and after reactions [2]. In addition, in order to explore the active sites and catalytic mechanisms of the catalysts, it is necessary to characterize the structure and functional groups of the catalysts before and after oxidation and catalysis using advanced in situ characterization techniques, such as Fourier transform infrared (FTIR) spectroscopy and in situ X-ray diffraction (single-crystal or powder). The following sections will further explain these characterization methods.

(A) In situ Single-crystal X-ray diffraction (SCXRD) [69]: This is the most commonly used method for analyzing crystal structures. It provides precise data related to crystal structure and can be used to explore specific open metal sites for catalysis. Software such as Shelxtl and Olex2 can be used to analyze the data and obtain visual representations of crystal structures. The use of the SCXRD technique on the synthesized isostructural frameworks [(Cd_4_O)_3_(hett)_8_] and [(Pb_4_O)_3_(hett)_8_] enables the observation of metal ion exchange (Figure 12).
(B) In situ Synchrotron radiation [70,71]: Synchrotron radiation is electromagnetic radiation emitted by charged particles moving at speeds close to the speed of light in a magnetic field. It has advantages such as a wide spectrum, high brightness, high collimation, and a clean light source. It also has pulse and time structure characteristics, making it a new light source for scientific research. Synchrotron radiation has high brightness, which allows for high-resolution (spatial resolution, angular resolution, energy resolution, and time resolution) experiments in materials science, physics, chemistry, and medicine.

**Figure 13:**
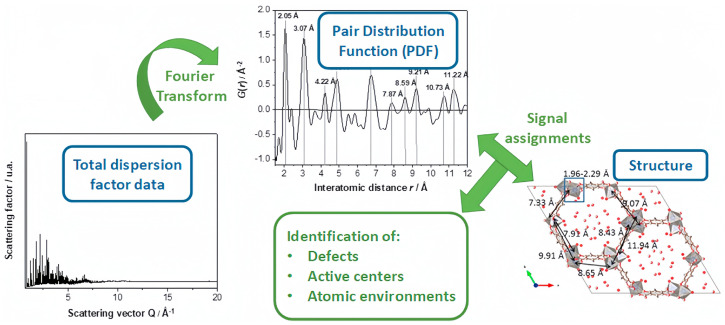
Schematic representation of obtaining the pair distribution function (PDF) and the information that arises from it, starting with the total dispersion factor data for conventional Zn−MOF−74. On the right, the structure of Zn−MOF−74 is shown (cif file: 1,494,750), along the c−axis, and near-neighbor distances [70]. Copyright 2021, Amsterdam, The Netherlands, Elsevier Inc.

**Figure 14:**
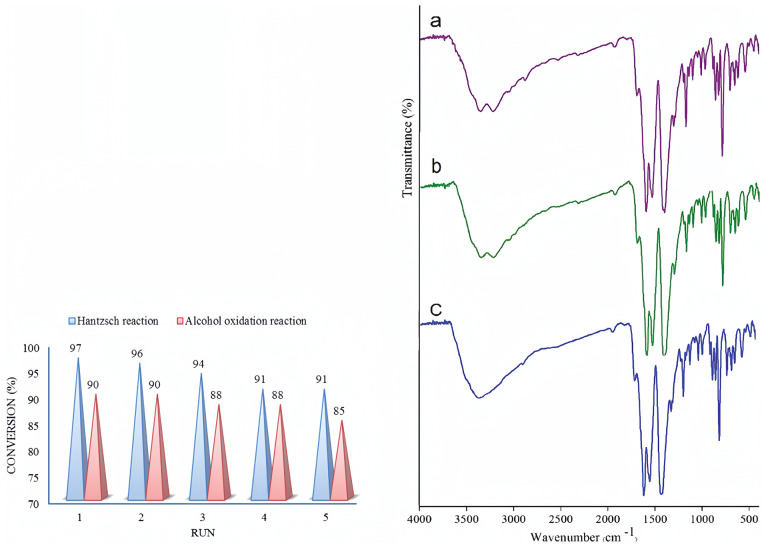
(**left**) Catalyst recyclability test for Hantzsch and alcohol oxidation reactions. (**right**) FTIR spectra of UoB−4: (**a**) fresh, (**b**) after the Hantzsch reaction; and (**c**) after alcohol oxidation [74]. Copyright 2020 © London, UK, The Royal Society of Chemistry and the Centre National de la Recherche Scientifique.

**Figure 15:**
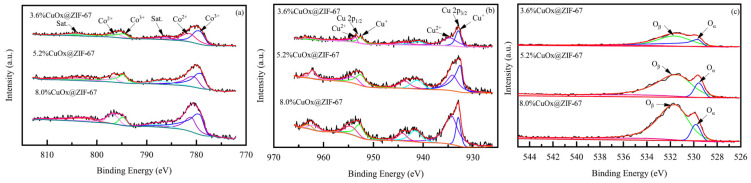
XPS spectra of Co 2p (**a**), Cu 2p (**b**), and O1s (**c**) of x%CuOx@ZIF-67 [76]. In figure (**a**), the pink and purple lines represent Co^2+^, while the green and blue lines represent Co^3+^. In figure (**b**), the pink and purple lines represent Cu^2+^, while the green and blue lines represent Cu^+^. In figure (**c**), the green line represents O_β_, while the blue line represents O_α_.

Through synchrotron radiation, total dispersion factor data of Zn-MOF-74 can be obtained, which can then be used to calculate the pair distribution function (PDF) and crystal defects, active centers, and atomic environments (Figure 13). This encompasses the crystal structure, making it easier to further understand the catalytic mechanism and active centers of MOF-based catalysts. Additionally, it facilitates the exploration of the correlation between crystal structure and catalytic performance.

(C) In situ Polycrystalline X-ray diffraction (PXRD) [69,72]: PXRD analysis only requires obtaining microcrystalline powder and preparing samples for testing, which is usually easier to obtain than the single crystals required for SCXRD. PXRD is a non-destructive analysis method based on X-ray diffraction, suitable for the qualitative or quantitative phase analysis of crystalline or amorphous materials. Similar to SCXRD, PXRD can obtain structural parameters of crystals and provide insights into changes in crystal structure during catalytic processes, which helps in understanding the relationship between catalytic mechanisms and performance and crystal structure. However, PXRD spectra suffer from peak overlap, provide less structural information compared to SCXRD, and are not suitable for directly determining unknown and complex crystal structures.
(D) In situ FTIR [73,74]: FTIR uses a continuous wavelength light source, and the interference pattern generated by infrared absorption of the sample can be transformed into a spectrum through the Fourier transform, allowing analysis of functional groups in the sample. FTIR has advantages such as fast scanning speed, high resolution, large photon flux, high sensitivity, wide spectral range, and high measurement accuracy. It can be used for qualitative and quantitative analysis of samples and is widely used in organic chemistry, biomedicine, materials science, and other fields. In situ FTIR can detect the chemical functional groups of MOF materials under different gas atmospheres. By comparing the FTIR spectra of fresh UoB-4 with those of UoB-4 subjected to the Hantzsch reaction and UoB-4 subjected to alcohol oxidation, the consumption and generation of chemical functional groups during the reaction can be determined, providing significant assistance in understanding the source of catalytic activity and facilitating the exploration of catalytic pathways (Figure 14).
(E) In situ XPS [75,76]: XPS uses X-rays to irradiate the surface of a material and measures the kinetic energy and quantity of electrons escaping from the material surface (usually within 10 nm) to obtain information about the elemental composition, content, and chemical state of the material surface. It can be used for the qualitative and quantitative analysis of samples. When different copper loading amounts of CuOx@ZIF-67 are analyzed using XPS (Figure 15), the impact of copper loading on the content and existing forms of cobalt, copper, and oxygen elements on the surface of CuOx@ZIF-67 can be understood. In situ XPS detection of MOF catalysts allows for the direct observation of catalyst restructuring during the catalytic process, including the generation and removal of catalytically active species in the reaction atmosphere, further exploring the catalytic origin and inferring the catalytic mechanism.

### 3.3. Evaluation of Catalytic Performance

After the preliminary structural determination of the catalyst, it is necessary to evaluate its actual catalytic performance, including its stability and catalytic activity, through experimental research and data analysis. The following section provides further explanation of the key data required for evaluating catalytic activity and the methods used for measurement.

(1) Conversion rate: The conversion rate refers to the proportion of reactants converted into products within a certain time period. It is an important indicator for evaluating catalyst performance as it directly reflects the efficiency and activity of the catalyst in the reaction process. A higher conversion rate indicates that the catalyst can more effectively promote the reaction while reducing side reactions and catalyst loss. The conversion rate can be determined using methods such as colorimetry [77,78], gas chromatography [79,80,81], and nuclear magnetic resonance [82].
(2) TOF [83]: TOF refers to the number of reactant molecules converted per active site on the catalyst per unit time. It is one of the key parameters for evaluating catalyst activity and efficiency. TOF can directly reflect the rate at which the reaction occurs on the catalyst per unit time, and it can also be used to assess the efficiency of the catalyst under specific reaction conditions. By comparing the TOFs of different catalysts, the most suitable catalyst can be selected, thereby improving the economics and sustainability of the reaction. Additionally, by measuring the TOF under different conditions, the influence of catalyst factors such as structure, composition, and morphology on its activity can be understood, guiding catalyst design and optimization. TOF can be inferred by measuring factors such as surface area, specific surface area, and active sites:
  (1) TOF=(r∗N)/(A∗t)
  where r is the reaction rate (mol/s), N is the number of active sites on the catalyst (mol), A is the surface area of the active sites (cm^2^), and t is the reaction time (s).

TOF can also be measured using analytical instruments such as mass spectrometers or gas chromatographs by measuring the flow rates of reactants and products [84,85]. The most commonly used method to determine the catalyst TOF is to apply the catalyst under specific reaction conditions, measure the reaction rate and product selectivity, and calculate the TOF [86,87].

(3) Selectivity: Catalyst selectivity refers to the ability of the catalyst to promote a specific reaction pathway among multiple possible pathways. Selectivity is crucial for achieving high conversion rates and the high purity of specific products. Catalysts with high selectivity can maximize the yield of the desired product, minimize the formation of by-products, and thus reduce the cost of waste treatment and separation steps, as well as minimize negative environmental impacts. Additionally, catalysts with high selectivity can provide high purity and selectivity for the target product, meeting market demands and quality standards. The selectivity of a catalyst can be evaluated by measuring the yield and selectivity for the target product using analytical methods such as gas chromatography, high-performance liquid chromatography [79,88], etc. It can also be studied by investigating the reaction mechanism and intermediates [79,89,90,91] providing a deeper understanding of selectivity. Furthermore, DFT calculations [43,92] can predict the energy barriers and reaction activity of different reaction pathways, facilitating the theoretical prediction of catalyst selectivity and the validation of reaction mechanisms.

### 3.4. Mechanistic Study

After evaluating the catalytic activity of a catalyst through specific experiments, it is necessary to infer the reaction mechanism, which is crucial for a fundamental understanding of reactions and the design of materials with improved performance. By utilizing in situ techniques such as FTIR [73,74] and XPS [75,76], information regarding the changes of functional groups and active species before and after the reaction can be obtained, facilitating a preliminary inference of the reaction mechanism. Additionally, DFT calculations serve as a tool to study multi-phase catalytic processes and their elementary steps at the atomic level, providing theoretical support and guidance for the proposed reaction mechanism.

DFT is a formulation of quantum theory developed based on the Thomas–Fermi theory. Traditional quantum theories consider the wave functions as the fundamental quantity for studying a system, while the main objective of DFT is to replace the wave function with the electron density, where the properties of the system are uniquely determined by its electron density distribution [93]. The landmark of modern density functional theory (DFT) was the proof of the Hohenberg–Kohn theorem in 1964 [94]. This theorem rigorously demonstrated that the ground state single-particle density of a many-particle system completely determines the system’s properties, and that the system’s energy as a function of the electron density distribution is minimized at the total ground-state energy of the system. Density functional theory provides a first-principles ab initio computational framework, describing a system using the electron density distribution function instead of wave functions, which greatly simplifies the description of multi-electron systems. Presently, DFT computational methods rely on solving approximate Kohn–Sham equations [95], with the computational complexity scaling cubically with the number of particles in the system. For large systems, it is much less computationally demanding compared to the Hartree–Fock–Roothaan (H-FR) method and can achieve a level of precision comparable to the MP2 method, making it widely applicable. DFT methods are capable of high-level calculations for systems containing 100–200 atoms (including several thousand valence electrons).

The effectiveness of DFT in addressing certain problems is attributed to its comprehensive consideration of electronic correlation effects within a system, i.e., the full account of electronic interactions. In contrast, ab initio methods only consider such effects in an averaged form (in the form of electron density). However, in reality, electronic correlation effects are the transient interactions of spin-opposite electrons, and the approximations in ab initio methods in this regard lead to some degree of inaccuracy in their computational results. DFT methods offer a level of computational precision equivalent to that of ab initio methods while being less time-consuming. Currently, advanced kinetic modeling allows for the conversion of basic rate constants obtained from DFT calculations into conversion rates under reaction conditions. By simulating the linear relationship between activation energy and descriptors (such as binding energy), the selectivity and activity of reactions can be theoretically predicted with knowledge of a small number of descriptors.

**Figure 16:**
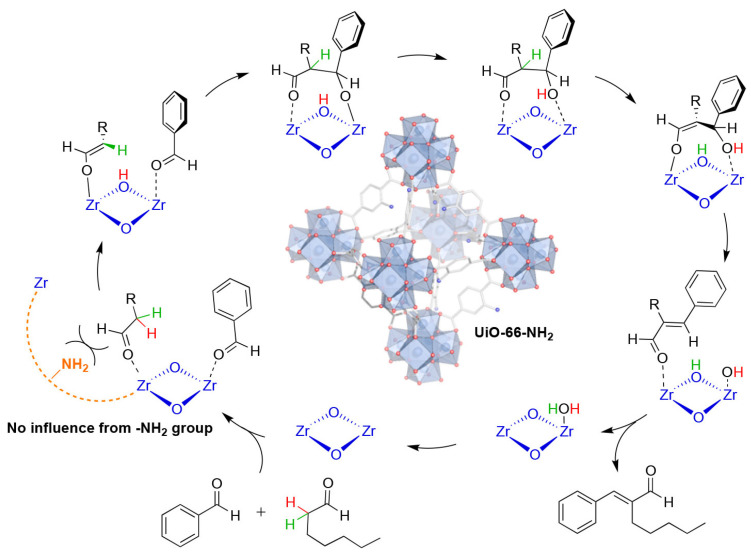
Revised mechanism of jasminealdehyde synthesis catalyzed by UiO-66/UiO-66-NH_2_ [10]. Copyright 2019, New York, NY, USA, American Chemical Society.

One major challenge in MOF-catalyzed reactions is the calculation of the catalytic mechanism, for which DFT provides a convenient tool. The research conducted by Moreno et al. [94] on Zr MOFs UiO-66 and UiO-66-NH_2_ provides a compelling illustration of the contribution of Density Functional Theory (DFT) to mechanistic understanding. Building upon experimental evidence suggesting that functionalization of terephthalate linkers with amino groups enhances the rate of jasminaldehyde synthesis, the authors utilized advanced DFT techniques and experimental validation to demonstrate that NH_2_ groups do not participate in the catalytic cycle (Figure 16). Instead, they facilitate substrate adsorption onto/into the MOF, thereby accelerating the initial rate. Nonetheless, the reaction proceeds through the same mechanism, and at later stages, the reaction rates remain similar, regardless of the presence of NH_2_ groups. Consequently, the authors identified proton transfer from the Zr-oxygen cluster to the carbonyl oxygen of the adsorbed aldol product as the rate-determining step, with hydrogen bonding of the amino groups being irrelevant to the reaction mechanism. A precise understanding of events within the pore and the interaction of reactants with the framework is crucial for designing improved catalysts, with DFT technology offering significant facilitation in this regard.

## 4. Summary and Outlook

Propylene is an important industrial raw material, and the production of propylene from abundant propane is a crucial direction in the industry. ODHP offers the advantage of lower operating temperatures compared to conventional propane dehydrogenation, thus reducing costs and potentially becoming a new pathway for industrial propylene production. In the context of the significant interest in the ODHP reaction, MOFs have shown unique advantages as catalysts and carriers for catalytically active species in the past seven years of research on ODHP catalysts. This article provides a comprehensive review of the recent progress in MOF-based catalysts for the ODHP reaction.

Although the development potential of MOF-based catalysts in the ODHP field has been demonstrated in recent years, there are still some challenges to overcome for their industrial application:

(1) Lack of sufficient research: While there have been numerous studies on the ODHP reaction, research on MOFs in the ODHP field is still not comprehensive enough. MOFs that exhibit excellent ODHP catalytic performance at low temperatures are yet to be discovered, and expanding the range of MOFs with ODHP catalytic activity is crucial for improving ODHP catalysts. The porous structure and diversity of metal centers and organic ligands in MOFs allow for the introduction of Lewis acidic metal ions and directional modifications of organic ligands to enhance catalytic performance, which is difficult to achieve with conventionally supported metal oxide catalysts.
(2) Lack of depth in the explanation of reaction mechanisms: While the research on MOFs in the ODHP field is still not enough, exploring the catalytic mechanism of MOFs is also a significant challenge. The diverse structures of MOF catalysts, consisting of various metal-organic frameworks, contribute to the complexity of understanding their catalytic mechanisms. The structural diversity of MOFs presents challenges in determining the structures of reaction transition states and intermediates. Additionally, the ODHP reaction involves high temperatures and the presence of oxygen, making it difficult to observe and determine the details of the reaction process in experiments. Furthermore, the structure and properties of active sites on the surface of MOF catalysts are often challenging to directly observe and determine, resulting in a lack of direct experimental evidence to prove the catalytic mechanism. However, a thorough understanding of the reaction mechanism is crucial for the study of MOF-catalyzed ODHP. To fully explore the catalytic mechanism of MOFs in ODHP, a combination of theoretical simulations (such as DFT) and advanced experimental techniques (such as in situ XAFS) are necessary.
(3) High costs and complex synthesis: MOF-based catalysts are typically in the form of powders or particles, and their recovery and recycling remain challenging. Moreover, MOFs often use complex organic ligands, which makes them more expensive than traditional metal oxide catalysts and less suitable for industrial applications. Additionally, the synthesis of MOFs is often performed on a laboratory scale, while industrial demands require large-scale synthesis. Scaling up the synthesis of MOFs requires overcoming challenges related to reaction condition control and crystal quality. Therefore, the prerequisite for the industrial application of MOF-based catalysts in ODHP is the synthesis of MOFs with simple, cost-effective, and easily scalable organic ligands.
(4) Stability: Although MOF-based catalysts exhibit excellent ODHP catalytic activity, they often cannot maintain high activity for prolonged periods. The challenge lies in developing structurally stable MOF-based catalysts that can maintain catalytic activity over extended periods in industrial settings and can be regenerated at low cost.

Despite the current lack of sufficient attention to the application of MOF-based catalysts in the ODHP reaction, their potential has been demonstrated in recent research over the past seven years. However, the industrial application of MOF-based catalysts for acrylic acid production still requires further research and the overcoming of various challenges. It is believed that with the diligent efforts of researchers, breakthroughs in the research on the ODHP reaction in the MOF field will be achieved. The development prospects of this work will make the propane dehydrogenation to propylene process more energy-efficient and, through future research, will achieve control over various aspects of MOF materials, thereby obtaining higher conversion rates and propylene selectivity. This advancement can drive the development of clean energy and a sustainable chemical industry, bringing about positive impacts on the environment and economy.

## Figures and Tables

**Table 1:** Catalytic effect of MOF-based catalysts and common catalysts on ODHP reaction.

Catalyst	Temperature/°C	Oxidizing Agent	Conversion Efficiency/%	Propylene Selectivity/%	Ref.	Remark
Co-AIM+NU-1000	230	O_2_	8.7	31.2	[11]	
CoAIM-Ni(II)SIM+NU-1000	230	O_2_	1.7	52.1	[12]	
MIL-100(Fe)	120	N_2_O	1.7	46.7	[13]	
SS-BNNS	490	O_2_	20.8	78.1	[14]	
MBON-2	490	O_2_	14.4	76.4	[2]	
6V/UiO-66	350	O_2_	17.08	49.7	[15]	
SS-BCNNSs	480	O_2_	34.9	92.6	[16]	photo-thermal
1.8V-SiO_2_	550	O_2_	12.7	57.1	[17]	
VA-5	500	O_2_	3.2	73	[18]	
3VTi	380	O_2_	3.8	73	[19]	
6V/Ti	400	O_2_	34.8	19.9	[20]	
F-PZr-V5.0	400	O_2_	4.0	63.6	[21]	
CA-P	550	O_2_	28.5	31.2	[22]	
NiO/CeO_2_	300	O_2_	12	60	[23]	
BNOH	540	O_2_	38.2	59.8	[24]	

**Table 2:** ODHP of MOFs-based catalysts.

Catalyst	Temperature/°C	Oxidizing Agent	Reactive Species	Conversion Efficiency/%	Propylene Selectivity/%	Ref.
Co-AIM+NU-1000	230	O_2_	Co(III)-O·	8.7	31.2	[11]
CoAIM-Ni(II)SIM+NU-1000	230	O_2_	Co(III)-O·	1.7	52.1	[12]
MIL-100(Fe)	120	N_2_O	Fe(IV)=O	1.7	46.7	[13]
SS-BNNS	490	O_2_	B-OH	20.8	78.1	[14]
MBON-2	490	O_2_	B=O	14.4	76.4	[2]
6V/UiO-66	350	O_2_	VO_x_	17.08	49.7	[15]

## Data Availability

Data are contained within the article.
